# Supplementary material for: Proteomic Profiling of Fallopian Tube-Derived Extracellular Vesicles Using a Microfluidic Tissue-on-Chip System
Source: Bioengineering (Basel). 2023 Mar 27;10(4):423. doi: 10.3390/bioengineering10040423 (PMC10135590; doi:10.3390/bioengineering10040423)
Supplement: Supplementary file 1 [file bioengineering-10-00423-s001.zip › bioengineering-2280625-supplementary.pdf]

bioengineering

Supplementary Information

Proteomic Profiling of Fallopian Tube-Derived Extracellular Vesicles Using a Microfluidic Tissue-on-Chip System

Didi Zha [1,†], Sagar Rayamajhi [2,†], Jared Sipes [2,†], Angela Russo [1], Harsh B. Pathak [2], Kailiang Li [1], Mihaela E. Sardiu [3,4], Leonidas E. Bantis [3], Amrita Mitra [2], Rajni V. Puri [2], Camille V. Trinidad [2], Brian P. Cain [5], Brett C. Isenberg [5], Jonathan Coppeta [5], Shannon MacLaughlan [6], Andrew K. Godwin [2,4,*] and Joanna E. Burdette [1,*]

[1] Department of Pharmaceutical Sciences, College of Pharmacy, University of Illinois at Chicago, Chicago, IL 60607, USA
[2] Department of Pathology and Laboratory Medicine, University of Kansas Medical Center, Kansas City, KS 66160, USA
[3] Department of Biostatistics and Data Science, University of Kansas Medical Center, Kansas City, KS 66160, USA
[4] Kansas Institute for Precision Medicine, University of Kansas Medical Center, Kansas City, KS 66160, USA
[5] Charles Stark Draper Laboratory, Cambridge, MA 02139, USA
[6] Department of Obstetrics and Gynecology, College of Medicine, University of Illinois at Chicago, Chicago, IL 60607, USA
* Correspondence: agodwin@kumc.edu (A.K.G.); joannab@uic.edu (J.E.B.)
† These authors contributed equally to this work.

Table of Contents:

1. Figure S1 ……………………………………………………………………2
2. Figure S2 ……………………………………………………………………3
3. Table S1 ……………………………………………………………………3
4. Table S2 ……………………………………………………………………4
5. Table S3 ……………………………………………………………………4

A

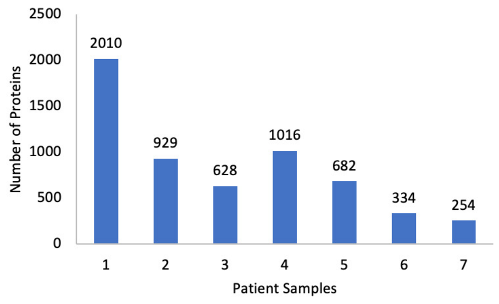

B

19 unique EV proteins from PREDICT hFTE

Gene ID	Protein name	Protein Accession number	N/7 samples
H4C1	Hisotne H4	P62805	7
H2BC12	Histone H2B type 1-K	O60814	7
H2BC11	Histone H2B type 1-J	P06899	7
H2AC4	Histone H2A type 1-B/E	P04908	7
H2AC20	Histone H2A type 2-C	Q16777	7
H2AZ1	Histone H2A.Z	P0C0S5	7
**H2AX**	**Histone H2AX**	**P16104**	**7**
H3-7	Histone H3-7	Q5TEC6	7
H3-3B	Histone H3	K7EK07	7
MACROH2A1	Core histone macro-H2A.1	O75367	7
MACROH2A2	Core histone macro-H2A.2	A0A7I2V412	7
ATP5F1B	ATP synthase subunit beta, mitochondrial	P06576	7
MGAM2	Probable maltase-glucoamylase 2	Q2M2H8	7
H1-2	Histone H1.2	P16403	5
H1-3	Histone H1.3	P16402	5
H1-4	Histone H1.4	P10412	5
RACK1	Receptor of activated protein C kinase 1	P63244	5
XP32	Skin-specific protein 32	Q5T750	5
ATP5F1A	ATP synthase subunit alpha	P25705	5

C

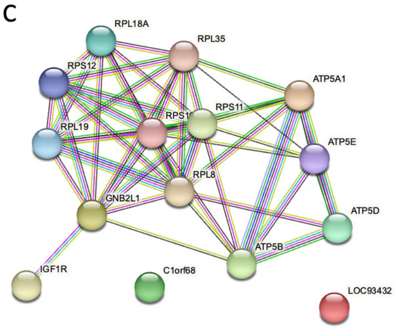

D

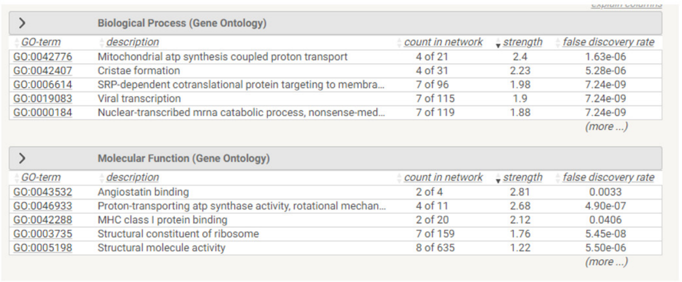

Figure S1. Proteomic profile of hFTE sEVs. (A) Number of protein identified in EVs derived from hFTE tissue explants from 7 different patients. (B) List of 19 unique EV proteins identified exclusively from our study. (C) Protein-protein interaction of 19 unique proteins. (D) Gene enrichment analysis showing biological process and molecular function associated with 19 unique proteins.

A

Term Description	Strength	False Discovery Rate	Matching proteins in your network (labels)
Leukocyte mediated immunity	0.85	6.39E-11	PYGB,PGRMC1,MPO,APCS,LYZ,CD81,RAB10,SERPINB12,SERPING1,HSPA6,RAB6A,HP,RAB5B,CD47,S100A7,S100A8,HRNR,RAP1A,HSPA1B,FLG2,HLA-A,CD44,RHOA,LAMP2,HLA-G,MGAM
Regulated exocytosis	0.83	6.39E-11	PYGB,PGRMC1,TIMP1,MPO,TUBA4A,LYZ,LGALS3BP,RAB10,SERPINB12,SERPING1,PLG,HSPA6,RAB6A,HP,CALM1,RAB5B,CD47,S100A7,S100A8,HRNR,RAP1A,HSPA1B,FLG2,CD44,RHOA,LAMP2,MGAM
Neutrophil degranulation	0.88	3.30E-09	PYGB,PGRMC1,MPO,LYZ,RAB10,SERPINB12,HSPA6,RAB6A,HP,RAB5B,CD47,S100A7,S100A8,HRNR,RAP1A,HSPA1B,FLG2,CD44,RHOA,LAMP2,MGAM
Cellular localization	0.44	1.51E-08	PYGB,PGRMC1,TIMP1,MPO,TUBA4A,YWHAH,LRPPRC,LYZ,ATP5B,LGALS3BP,CD81,TUBB4A,YWHAE,RAB10,SERPINB12,SERPING1,THY1,RAB6B,TUBA1C,RPS9,PLG,RPL15,HSPA6,BANF1,RAB6A,PPP2R1A,RPS2,HP,CALM1,RAB5B,CD47,S100A7,S100A8,HRNR,RAP1A,H2AFY2,HSPA1B,FLG2,FLOT2,ATP5A1,RPS18,CD44,RHOA,LAMP2,H2AFY,RAN,MGAM
Establishment of localization in cell	0.48	3.48E-08	PYGB,PGRMC1,TIMP1,MPO,TUBA4A,YWHAH,LRPPRC,LYZ,ATP5B,LGALS3BP,YWHAE,RAB10,SERPINB12,SERPING1,RAB6B,TUBA1C,RPS9,PLG,RPL15,HSPA6,BANF1,RAB6A,RPS2,HP,CALM1,RAB5B,CD47,S100A7,S100A8,HRNR,RAP1A,HSPA1B,FLG2,FLOT2,ATP5A1,RPS18,CD44,RHOA,LAMP2,RAN,MGAM

B

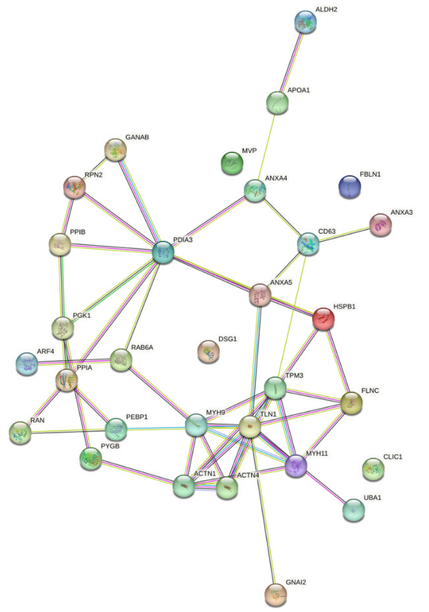

Figure S2. Pathway and protein interaction analysis of sEV proteins exclusively detected in human or serous tubal intraepithelial carcinoma (STIC). (A) Go term analysis of 119 human unique EV proteins. (B) Protein-protein interaction networks of 32 sEV proteins exclusively detected in STIC**.**

Category	CA#	Age	Reason for surgery	Uterine diagnosis	FT Dx
Sample 01	461_27257	65	Endometrial Cancer	Adenosarcoma	Benign
Sample 02	461_27262	57	Endometrial Cancer	Endometrial adenocarcinoma	Benign
Sample 03	461_27266	47	EIN	Endometrial intraepithelial neoplasia (EIN)	Benign
Sample 04	461_27268	63	Endometrial Cancer	Endometrial adenocarcinoma	Benign
Sample 05	461_27273	60	Endometrial Cancer	Endometrial adenocarcinoma	Benign
Sample 06	461_27277	64	Endometrial Cancer	Endometrial adenocarcinoma	Benign
Sample 07	461_27283	60	EIN	Atrophic endometrium	Benign

Table S1. Details of patient used as the source of fallopian tube epithelial tissue sEV in the proteomics.

	Bovine	Procine	Feline	Human
Bovine	1	0.068053993	0.108235294	0.198039216
Porcine	0.068053993	1	0.088515834	0.04386423
Feline	0.108235294	0.088515834	1	0.084084084
Human	0.198039216	0.04386423	0.084084084	1

Table S2. Jaccard similarity index of oviduct EV proteins detected in domestic, farm animals and human.

SN	Gene ID	Protein name
1	CD59	CD59 glycoprotein
2	CD81	CD81 antigen
3	CD9	CD9 antigen
4	CD47	Leukocyte surface antigen CD47
5	NT5E	5'-nucleotidase
6	ITGB1	Integrin beta
7	HLA-DRA	HLA-DRA
8	LAMP2	Lysosome-associated membrane glycoprotein 2
9	THY1	Thy-1 antigen
10	CD44	CD44 antigen
11	CD63	CD63 antigen
12	HLA-A	HLA class I histocompatibility antigen, alpha chain A
13	SLC2A1	Solute carrier family 2, facilitated glucose transporter member 1
14	DPP4	Dipeptidyl peptidase 4
15	HLA-G	HLA class I histocompatibility antigen, alpha chain G

Table S3. The list of 15 membrane proteins out of 61 common genes/proteins between hFTE transcriptome and hFTE sEV proteomics.
